# Alpha-1-antichymotrypsin: a potential inducer for epithelial-mesenchymal transition in lupus nephritis

**DOI:** 10.1515/biol-2025-1319

**Published:** 2026-05-20

**Authors:** Xiaoyan Huang, Cuijuan Zhang, Ming Yang, Xiaoshu Dong, Hui Zhang, Xingjiao Liu, Yi Jiang, Yongfei Wang, Yushuang Wei, Bing Yuan, Meiying Wang

**Affiliations:** Department of Nephrology, Peking University Shenzhen Hospital, Shenzhen, Guangdong Province, 518036, China; Department of Cardiovascular Surgery, First Center of 301 Chinese PLA General Hospital, Beijing, 100853, China; Department of Otolaryngology, Shenzhen People’s Hospital, Shenzhen 518020, China; Department of Rheumatology and Immunology, Shenzhen Second People’s Hospital, The First Affiliated Hospital of Shenzhen University, Shenzhen, Guangdong Province, 518055, China; Department of Laboratory Medicine, Shenzhen Second People’s Hospital, The First Affiliated Hospital of Shenzhen University, Shenzhen, Guangdong Province, 518020, China; School of Medicine and Warshel Institute for Computational Biology, Chinese University of Hong Kong, Shenzhen, Guangdong, China; Department of Paediatrics and Adolescent Medicine, University of Hong Kong, Hong Kong, China; Songshan Lake Materials Laboratory, Dongguan, Guangdong Province, 523808, China

**Keywords:** systemic lupus erythematosus, lupus nephritis, renal fibrosis, alpha-1-antichymotrypsin, epithelial-mesenchymal transition

## Abstract

Lupus nephritis (LN) is a severe complication of systemic lupus erythematosus and represents a major risk factor for mortality. The critical pathological process affecting the renal parenchyma is fibrosis, which ultimately progresses to end-stage kidney disease. Identifying the molecular mediators of fibrosis is crucial for developing novel therapeutic strategies for lupus patients. Herein, we demonstrate that alpha-1-antichymotrypsin (AACT) is a critical inducer of the epithelial-mesenchymal transition (EMT) in HK-2 cells. Immunohistochemical analysis reveal that AACT expression progressively increases with advancing LN class. We next generate AACT knockout (AACT^KO^) and overexpressing (AACT^OE^) HK-2 cell lines. Our results indicate that AACT levels are positively correlated with cellular migration, the expression of migration-associated proteases, and collagen secretion in HK-2 cells. Notably, AACT inhibits the expression of the epithelial marker E-cadherin while promoting that of the mesenchymal marker α-SMA. Therefore, these findings suggest that AACT may contribute to renal fibrosis via triggering EMT, thereby promoting cell migration and collagen accumulation within renal tissues. Our study identifies AACT as a potential therapeutic target to counteract LN-associated renal fibrosis.

## 1 Introduction

Systemic lupus erythematosus (SLE) is a severe autoimmune disease characterized by the presence of autoantibodies against several self-antigens [1], [2], [3]. Its heterogeneity includes diverse clinical presentations, the potential involvement of multiple organ systems, and the wide range of immunoserological test results [4]. Lupus nephritis (LN) is a major cause of morbidity and mortality, affecting approximately 40 % of adults and 80 % of children with SLE [5], 6]. The current standard of care includes an induction therapy with high doses of immunosuppressive agents and glucocorticoids followed by maintenance for several years [7], 8]. However, even in clinical trials, only 30–50 % of patients achieve remission and 10–20 % progress to end stage renal disease within a decade from diagnosis [9].

LN is primarily characterized by glomerulonephritis, often accompanied by damage to the renal tubular, interstitial, and capillary structures [10]. Numerous cytokines, such as monocyte chemoattractant protein-1, tumor necrosis factor-α (TNF-α), and interleukin-6, have been identified as LN markers or caused long-term renal inflammation [11], 12]. This inflammation-mediated kidney injury eventually leads to fibrosis. According to clinical statistics, renal fibrosis affects up to 50 % of LN patients and is closely associated with the prognosis of this disease [13]. Epithelial-mesenchymal transition (EMT) has been widely recognized as a critical mechanism contributing to LN-associated renal fibrosis [14]. During LN development, EMT occurrence of intrinsic renal epithelial cells contributes to parenchymal cell dysfunction, which is common in early stages of chronic fibrosis caused by various kidney diseases. There is strong evidence that EMT correlates with poor renal outcomes [15]. Notably, EMT reflects exposure to a profibrotic milieu at an early and potentially reversible stage [16]. Therefore, inhibition of EMT process during LN progression represents a potential therapeutic approach against fibrosis.

Alpha-1-antichymotrypsin (AACT), a secretory serine protease inhibitor, inhibits the activity of several serine proteases such as cathepsin G and chymotrypsin [17]. Importantly, AACT has been implicated in the pathogenesis of numerous human diseases including chronic obstructive pulmonary disease [18], Alzheimer’s disease [19], and cystic fibrosis [20]. In patients with LN, urinary AACT levels are associated with disease activity [21]. These levels are higher in SLE patients with active renal disease than those in active nonrenal or inactive SLE patients, suggesting that AACT is involved in LN pathogenesis but not as a general marker for inflammation. Urine AACT levels also decreased after successful treatment at 6 and 12 months in LN patients. Microarray studies have shown that AACT is transcriptionally upregulated during renal injury. Both the adenine diet model of chronic kidney injury and the renal ischemia reperfusion injury model of acute kidney injury displayed AACT upregulation in tubular cells [22]. However, the pathological role of AACT in renal fibrosis remains unclear. In this study, we aimed to investigate the expression profile of AACT in the kidney biopsies from different classes of LN and assess its contribution to EMT during renal fibrosis by a series of *in vitro* assays.

## 2 Materials and methods

### 2.1 Patients and samples

Archived formalin-fixed and paraffin embedded renal specimens were obtained from Peking University Shenzhen Hospital. All patients fulfilled the pathological classification of LN revised by the International Society of Nephrology (ISN) and the Society of Renal Pathology (RPS) in 2003 (2003 ISN/RPS). Patients diagnosed with mixed connective tissue disease/overlap syndrome, known HIV, positive hepatitis B/C or uncontrolled diabetes mellitus were excluded from this study. Surveillance transplant kidney biopsies without pathology were used as normal controls (NCs). As a retrospective, case-controlled study of renal biopsies, informed consent exemption and ethical approval was granted by the Ethics Committee of Peking University Shenzhen Hospital (No. 2021-038). Blocks of 48 renal biopsies including 42 LN patients and 6 NCs met the above inclusion and exclusion criteria. Demographic features and histopathological findings of the patients were described in Supplementary Table 1.

**Informed consent:** Informed consent has been obtained from all individuals included in this study.

**Ethical approval:** The research related to human use has been complied with all the relevant national regulations, institutional policies and in accordance with the tenets of the Helsinki Declaration, and has been approved by the Ethics Committee of Peking University Shenzhen Hospital (No. 2021-038).

### 2.2 Immunohistochemistry for AACT

Renal biopsies were fixed in 4 % paraformaldehyde and sectioned into 5 μm-thick slices. Sections were incubated overnight at 40 °C, dewaxed, and then subjected to antigen retrieval by autoclaving at 100 °C for 3 min in a citric acid buffer (pH = 6.0). Endogenous peroxidase activity was quenched with 0.3 % H_2_O_2_ for 30 min. Nonspecific binding was blocked with normal goat serum for 1 h, followed by an overnight incubation at 4 °C with a specific primary antibody against AACT (SAB, USA). Subsequently, the sections were incubated with a secondary antibody for 30 min at 37 °C. AACT protein expression was visualized using 3,3′-diaminobenzidine. Images were captured using a light microscope (Zeiss Corporation, Germany).

### 2.3 AACT knockout and overexpression in HK-2 cells

Small guide RNAs (sgRNA) targeting human AACT (AACT-Cas9-sgRNA1: cgg​att​agc​ctc​cgc​caa​cg, AACT-Cas9-sgRNA2: tct​gct​gga​cag​gtt​cac​gg) were inserted into the PmaxGFP-puro plasmid. The plasmid (2 μg) was then electroporated using an SE-DN100 system into HK-2 cells at 70 % confluence. Subsequently, GFP-positive cells were sorted by FACS and cultured in a selection medium containing 2 μg/mL puromycin (Thermo Scientific, USA). Successful generation of stable AACT knockout (AACT^KO^) HK-2 cells was verified by qPCR assay. Control cells were transfected with a PmaxGFP-puro plasmid encoding a nonsense sgRNA.

The complementary DNA (cDNA) of AACT was inserted into the hU6-MCS-Ubiquitin-EGFP-IRES-puromycin vector to generate an AACT expression vector. This construct was co-transfected with lentiviral packaging plasmids into HEK-293 cells to produce viral particles. Following filtration and enrichment to a titer of 10^8^ TU/mL, the viral particles were added to transduce HK-2 cells. The infected cells were cultured for five to six additional passages in a selection medium containing 5 μg/mL puromycin. Successful AACT overexpression (AACT^OE^) in HK-2 cells was verified by qPCR assay.

### 2.4 qPCR assay

Total RNA was isolated using the RNeasy Mini kit (Qiagen, USA). cDNA was synthesized using the Omniscript RT Kit (Qiagen, USA), and then subjected to qPCR analysis. Real-time monitoring of the following genes was performed with TaqMan probes (Life Technologies, USA) on an ABI Prism 7900 system (Applied Biosystems, USA). The primer sequences were as follows:

AACT-Forward: 5′-TGC​CAG​CGC​ACT​CTT​CAT​C-3′,

AACT-Reverse: 5′-TGT​CGT​TCA​GGT​TAT​AGT​CCC​TC-3′;

GAPDH-Forward: 5′-CAT​CAT​CCC​TGC​CTC​TAC​TGG-3′,

GAPDH-Reverse: 5′-GTG​GGT​GTC​GCT​GTT​GAA​GTC-3′.

### 2.5 Transwell migration assays

1 × 10^4^ of HK-2 cells with varying AACT expression were plated in the upper chamber of a transwell plate with serum-free medium, while the lower chamber contained 40 % FBS medium. After a 24 h incubation, the medium was removed, and the cells were washed three times. Non-invading cells remaining in the upper chamber were removed by scrubbing with cotton-tipped swabs. The invading cells on the underside of the membrane were then fixed with 4 % paraformaldehyde for 20 min and subsequently stained with crystal violet (Coolaber, China) for 20 min. Images of all fields within each chamber were captured, and the number of invading cells was quantified using a High-Content Screening Applications system (Thermo Scientific, USA).

### 2.6 In vitro wound healing assay

Wound healing assay was performed using control (AACT^Ctrl^), AACT^KO^, and AACT^OE^ HK-2 cells. Cells were seeded at a density of 2 × 10^5^ cells per well in 24-well plates and cultured to reach 80 % confluence. The confluent cell monolayers were then washed with 1 % PBS. A scratch was introduced through the center of each well using a yellow pipette tip to create a wound approximately 0.5 mm in width. Cell residues in the plate were washed with 1 % PBS, then incubated for 24 h at 37 °C and photographed under an inverted microscope.

### 2.7 Immunocytochemistry assay

AACT^Ctrl^, AACT^KO^, and AACT^OE^ cells were first plated in confocal dishes for 12 h and then incubated with 10 ng/mL TGF-β for another 24 h. The cells were then washed with 1 % PBS and incubated for 24 h with primary antibodies of E-cad and α-SMA (Abcam, UK). Subsequently, the cells were incubated with a green fluorescence-conjugated secondary antibody (Abcam, UK) and observed using a confocal laser scanning microscope (OLYMPUS, Japan).

### 2.8 Western blots

Western blots were performed according to the literature [23]. Cells were lysed in ice-cold protein lysis buffer. Proteins (30 μg) were separated by SDS-PAGE, transferred to a PVDF membrane and incubated with anti-MMP2 (Abcam, #ab92536, 1:2000 dilution), anti-MMP9 (Abcam, #ab58803, 1:500 dilution), anti-Ecad (Abcam, #ab40772, 1:1,000 dilution), and β-actin (Cell Signaling Technology, #4967, 1:1,000 dilution), followed by incubation with horseradish peroxidase-conjugated secondary antibody (Cell Signaling Technology, USA). The signals were visualized using an ECL detection kit (Thermo Scientific, USA). The gray values were calculated using the ImageJ software.

### 2.9 The detection of collagen I and IV by ELISA

Corresponding ELISA kits (SAB, USA) were used to detect collagen I and IV secreted by HK-2 cells into the supernatants. The assay was performed in duplicate according to the manufacturer’s protocol. Briefly, AACT^Ctrl^, AACT^KO^, and AACT^OE^ cells were cultured with or without 10 ng/mL TGF-β stimulation for 72 h at 37 °C. Supernatants were then collected for the detection of collagen I and IV. The detection process was performed as described previously [24].

### 2.10 Statistical analysis

Statistical analysis was performed using GraphPad Prism 8 software (Graphpad Software, USA). Unpaired two-tailed Student’s t-test was used to assess the difference between any two groups. One- or two-way ANOVA test was used for multiple comparisons tests. Error bars were indicated as the mean ± standard deviation of at least three independent experiments unless otherwise specified. The differences were considered statistically significant if *p* < 0.05. *p < 0.05, **p < 0.01, and ***p < 0.001.

## 3 Results

### 3.1 AACT is overexpressed in LN kidney biopsies across different classes

**Figure 1:**
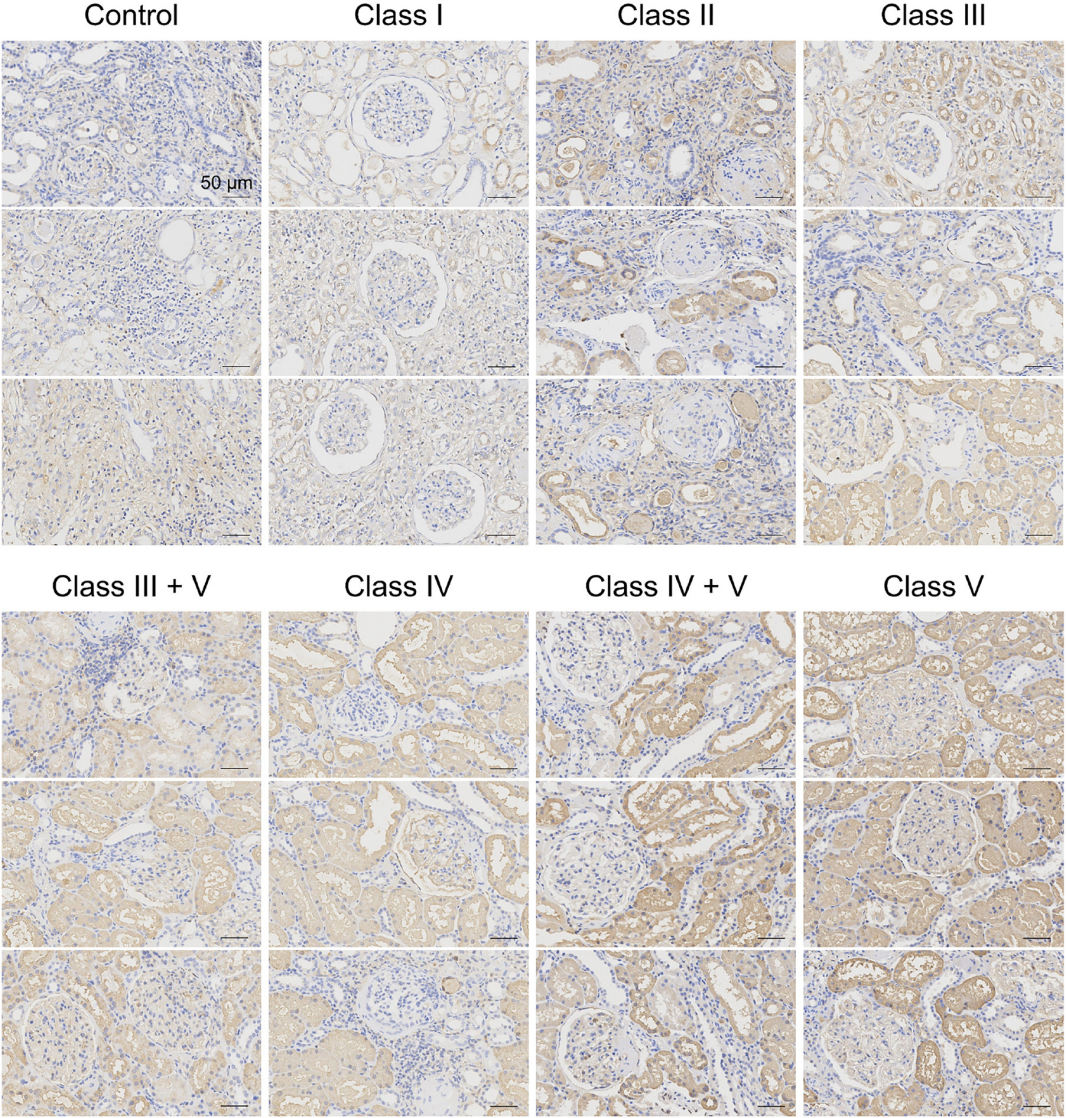
High expression of AACT in renal tissues from LN patients with different classes. Immunohistochemical assays for AACT in renal tissues (class I to class V) from LN patients and NCs samples. Scale bar is 50 μm.

To investigate the expression profile of AACT and its correlation with LN development, clinical kidney samples were collected from 6 NCs and 42 LN patients with class I (*n* = 6), class II (*n* = 6), class III (*n* = 6), class III + V (*n* = 6), class IV (*n* = 6), class IV + V (*n* = 6), and class V (*n* = 6). In all classes of LN patients, significantly higher levels of AACT were observed in renal tissues (Figure 1). In NCs tissues, weak staining for AACT was seen in the cytoplasm and brush border of proximal tubules and peritubular capillaries, whereas only minimal staining was observed in glomerular endothelial cells. Compared to NCs, the expression of AACT gradually increased as LN class progressed. Intense AACT staining was observed in the cytoplasm and brush border of proximal tubule and some distal tubule epithelial cells in the kidney tissues of LN patients with class I, II, III, III + V, IV, IV + V and V. There was also evidence of AACT staining in the tubular lumen and peritubular capillaries. However, within the glomeruli, only weak staining was observed in podocytes, glomerular endothelial cells, and parietal epithelial cells across all LN classes. The preferential overexpression of AACT in proximal tubule cells compared to other renal cell types suggests that AACT may play an important role in promoting renal pathology in LN via effects on tubular epithelial cells.

### 3.2 AACT expression is positively correlated with HK-2 cells migration

Immunostaining results of LN renal tissues indicated that AACT expression may contribute to the progression of LN. The early event in LN development is EMT progression in renal epithelial cells, which leads to renal fibrosis. Thus, inhibiting or reversing EMT could serve as an effective treatment for renal fibrosis. To investigate whether AACT regulates the migration ability of renal epithelial cells, we achieved knockout and overexpression of AACT in HK-2 cells via CRISPR-Cas9 and transfection techniques. Successful integration of both sgRNA plasmids into HK-2 genomic DNA was observed with increased fluorescence intensity correlated with transfection duration (Supplementary Figure S1a, b). Notably, PCR assay showed the oligonucleotide insertion in two sgRNA-transfected cells. AACT-Cas9-sgRNA2 exhibited a higher transfection efficiency than that of AACT-Cas9-sgRNA1 (Supplementary Figure S2). Thus, we selected ACT-Cas9-sgRNA2 for subsequent AACT knockout in HK-2 cells.

**Figure 2:**
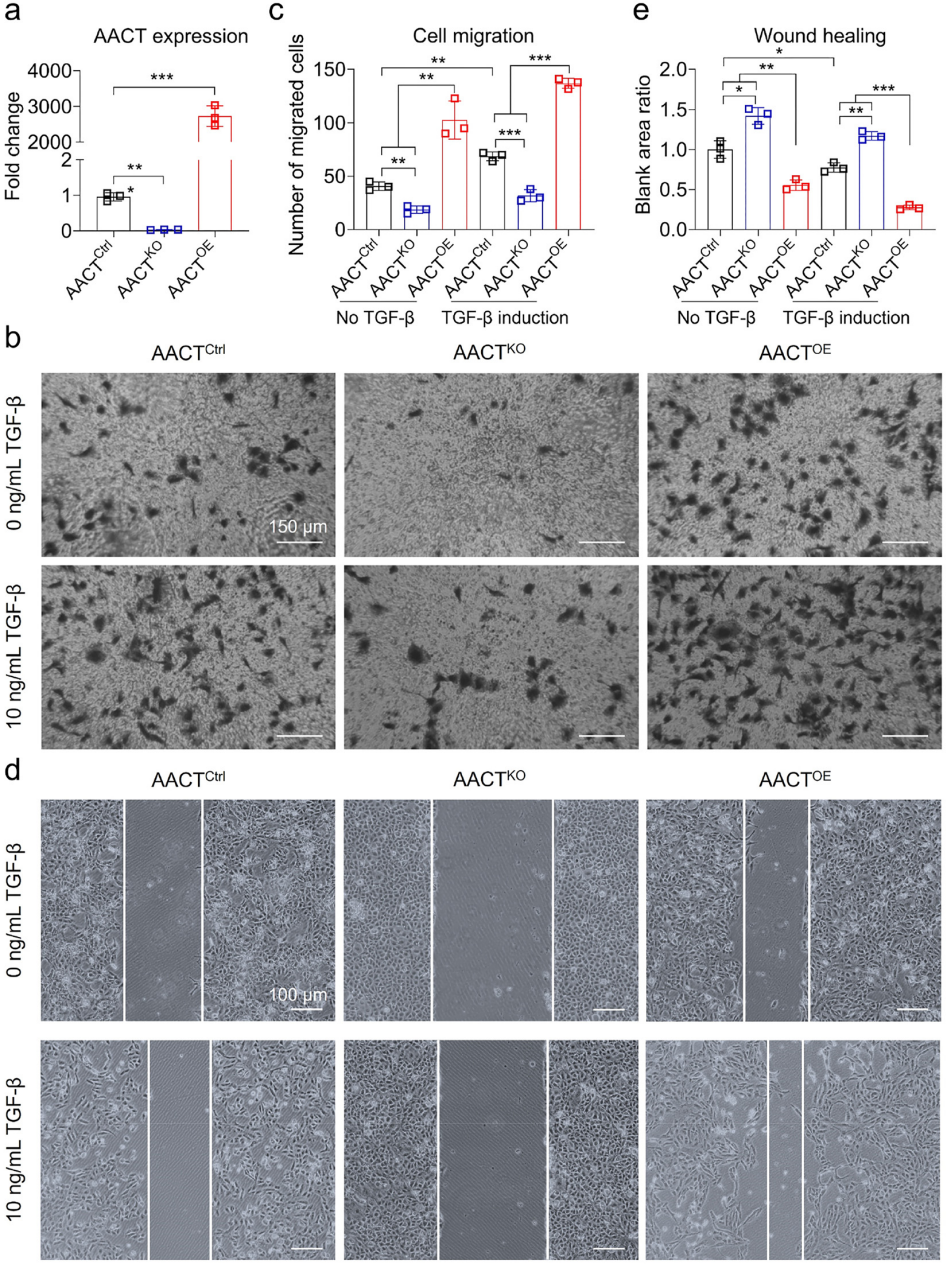
AACT promotes the migration of HK-2 cells. (a) qPCR assay was utilized to evaluate the efficiency of AACT^KO^ and AACT^OE^ in HK-2 cells. (b–c) Transwell assay was performed to evaluate the migratory ability of AACT^Ctrl^, AACT^KO^ and AACT^OE^ HK-2 cells with or without 10 ng/mL TGF-β induction. Scale bar is 150 μm. (d–e) In vitro cell wound healing assay showed that AACT promotes HK-2 migration. Scale bar is 100 μm. *p < 0.05, **p < 0.01, ***p < 0.001.

To quantify the knockout and overexpression of AACT in HK-2 cells, qPCR assay for AACT expression were conducted. The results showed that the AACT gene was significantly downregulated in AACT^KO^ cells, while it was overexpressed in AACT^OE^ cells (Figure 2a). Transwell assay was subsequently conducted to evaluate the association between AACT expression and the migratory capacity of HK-2 cells. In the absence of TGF-β induction, the number of migrated AACT^KO^ cells decreased compared with AACT^Ctrl^ cells. Conversely, the acount of migrated AACT^OE^ cells increased significantly compared with AACT^Ctrl^ cells. To further investigate AACT-driven migration of HK-2 cells, TGF-β was employed to stimulate these cells to mimic the process of epithelial EMT. It was observed that 10 ng/mL TGF-β could significantly induced the occurrence of EMT process. At this concentration, the number of migrated cells in each group increased. The migration ability of HK-2 cells was positively correlated with the expression level of AACT regardless of whether it was stimulated by TGF-β or not (Figure 2b and c). To reconfirm this finding, we performed *in vitro* wound healing assays. The results revealed that the expression level of AACT was positively correlated with the migration ability of HK-2 cells (Figure 2d and e). Without TGF-β induction, AACT^KO^ cells exhibited virtually absent migration capacity, whereas the migration ability of HK-2 cells increased correspondingly with increasing AACT expression. With 10 ng/mL TGF-β induction, the positive correlation between AACT expression and the migration ability of HK-2 cells was further expanded. Compared to AACT^Ctrl^ and AACT^OE^ cells, the migration ability of AACT^KO^ cells was the weakest. Importantly, AACT overexpression markedly promoted the migration of HK-2 cells. Thus, AACT is a critical inducer that determines the migration ability of HK-2 cells.

### 3.3 AACT promotes the expression of EMT-associated proteins in HK-2 cells

**Figure 3:**
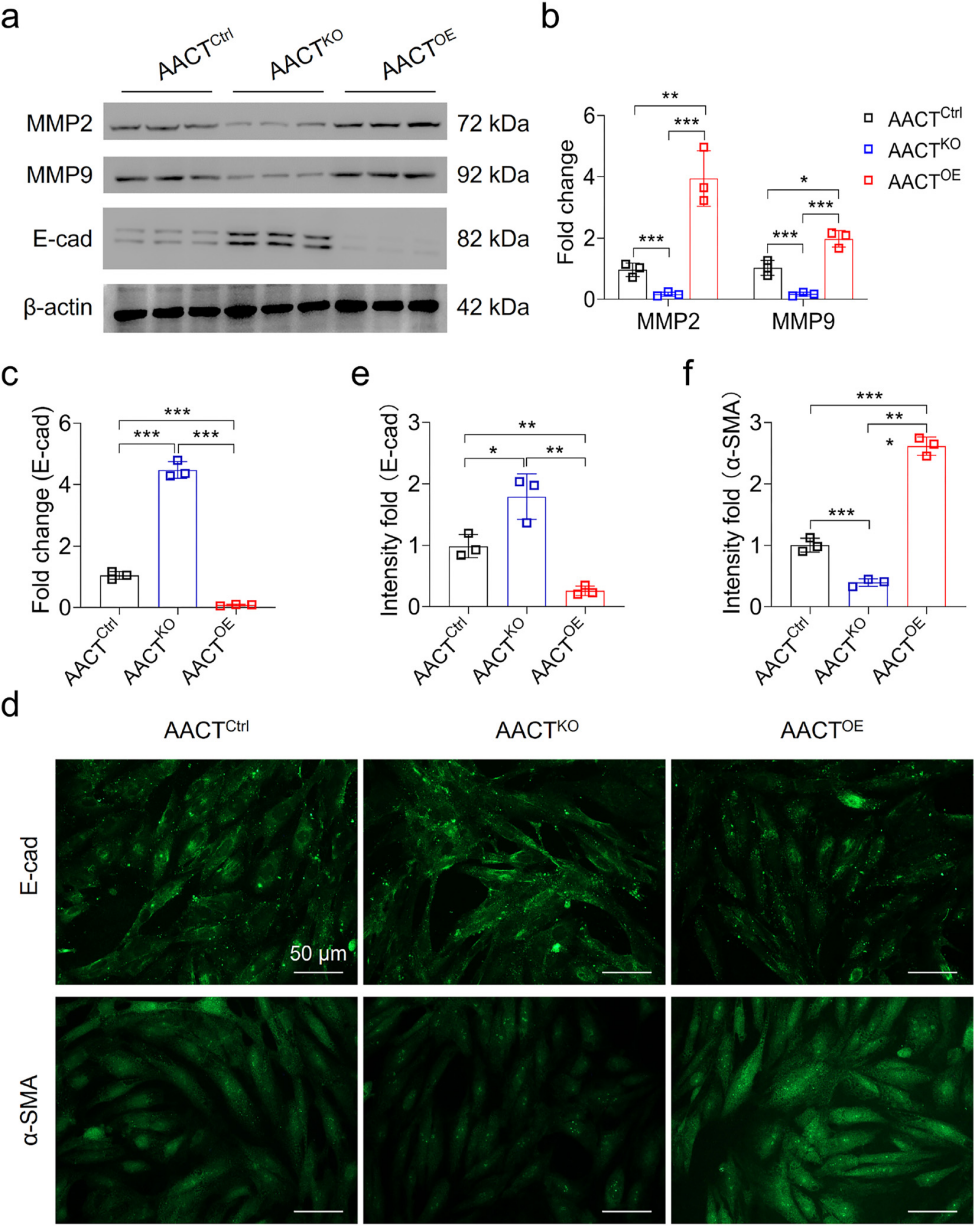
AACT mediates the expression of EMT-associated protein. (a) Western blots showed that the expression of MMP2, MMP9, and E-cad was regulated by AACT in HK-2 cells. (b–c) Statistics of AACT-mediated the expression of MMP2, MMP9, and E-cad in HK-2 cells. (d) The immunocytochemistry assay showed that EMT markers E-cad and α-SMA were regulated by AACT in HK-2 cells. Scale bar is 50 μm. (e–f) Statistics of the fluorescence intensity fold changes of E-cad and α-SMA in AACT^KO^, AACT^Ctrl^, and AACT^OE^ cells. *p < 0.05, **p < 0.01, ***p < 0.001.

The dynamic expression of MMP2 and MMP9, E-cad, and α-SMA serve as the indicators of EMT process [25], 26]. We next evaluated whether AACT regulates the expression of these EMT associated proteins in HK-2 cells. In western blot analysis, we observed a significant downregulation of MMP2 expression in AACT^KO^ cells compared to AACT^Ctrl^ cells. Conversely, MMP2 was only marginally upregulated in AACT^OE^ cells relative to AACT^Ctrl^ cells. Moreover, MMP9 was elevated in AACT^OE^ cells compared to both AACT^Ctrl^ and AACT^KO^ cells. These findings highlighted the importance of the AACT/MMP axis in determining the migratory capacity of HK-2 cells (Figure 3a and b). E-cad, a typical epithelial marker, was significantly downregulated in AACT^OE^ cells compared to AACT^Ctrl^ and AACT^KO^ cells (Figure 3a, c, and Supplementary Figure S3).

**Figure 4:**
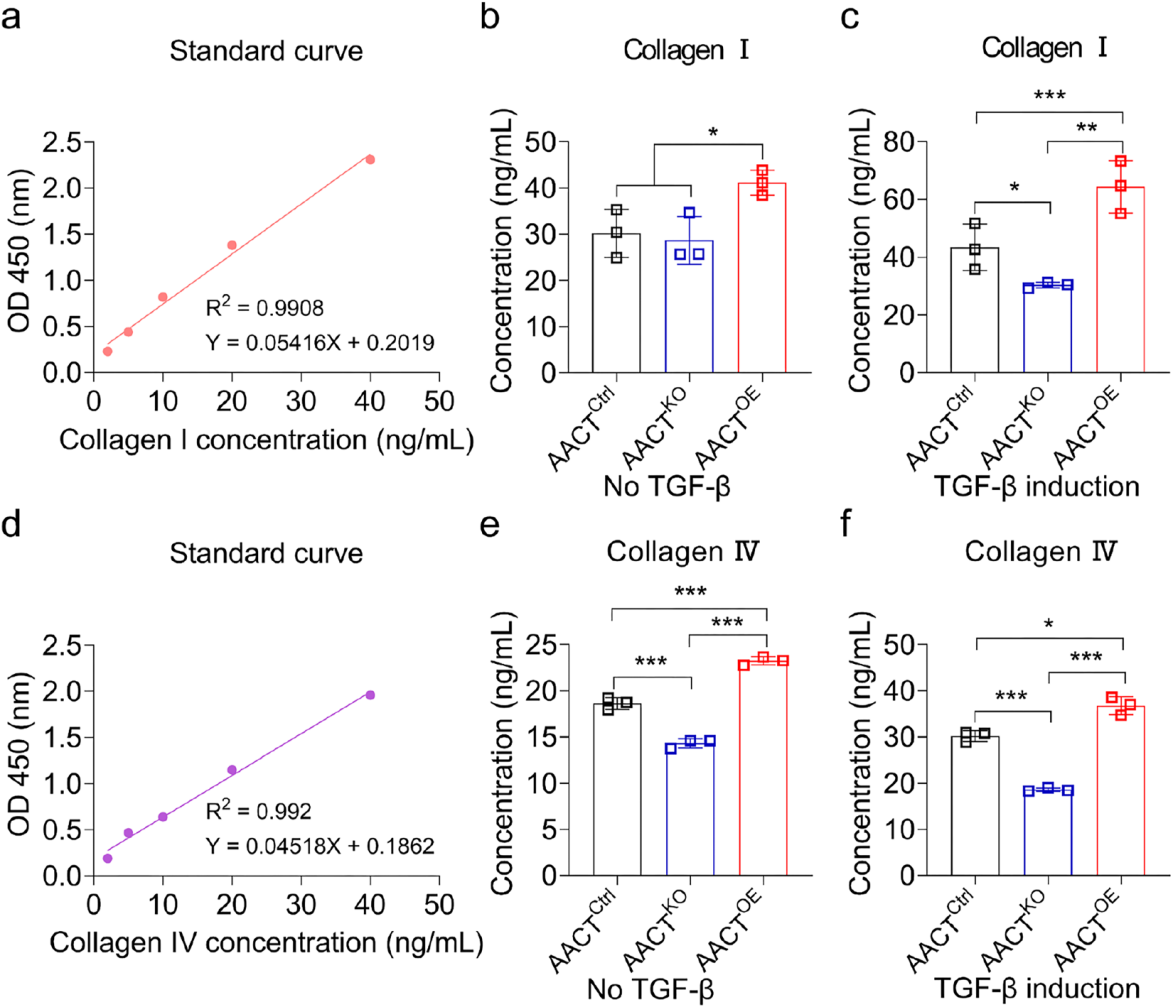
AACT enhances the secretion of collagen I and IV in HK-2 cells. (a, d) The standard curves of collagen I and IV in ELISA assays. (b, e) ELISA detection of collagen I and IV in the supernatant of AACT^KO^ and AACT^OE^ HK-2 cells without TGF-β induction. (c, f) ELISA detection of collagen I and IV in the supernatant of AACT^KO^ and AACT^OE^ HK-2 cells with 10 ng/mL TGF-β induction. *p < 0.05, **p < 0.01, ***p < 0.001.

To further elucidate the relationship between AACT level and EMT occurrence, immunocytochemistry assay was performed to evaluate the expression of E-cad in HK-2 cells. As depicted in Figure 4e and f, E-cad was upregulated in AACT^KO^ cells relative to AACT^Ctrl^ cells, while its expression was significantly downregulated in AACT^OE^ cells (Figure 3d and e). In contrast, the mesenchymal marker α-SMA was evidently downregulated in AACT^KO^ cells and upregulated in AACT^OE^ cells compared with AACT^Ctrl^ cells (Figure 3d, f). These results indicate that the increased AACT expression is a critical inducer for EMT occurrence in HK-2 cells.

### 3.4 AACT enhances the secretion of type I and IV collagen in HK-2 cells

Renal fibrosis is characterized by the accumulation of abnormal extracellular matrix components, including type I and IV collagen [27]. To determine whether AACT regulates collagen secretion in HK-2 cells, we collected the culture media from AACT^Ctrl^, AACT^KO^, and AACT^OE^ cells and measured the levels of collagen I and IV using ELISA assays. The results presented standard curves for collagen I and IV with R^2^ values of 0.9908 and 0.992 (Figure 4a and d). In the absence of TGF-β induction, the concentration of collagen I was significantly increased in the culture medium of AACT^OE^ cells compared to that of AACT^Ctrl^ cells. However, AACT^KO^ failed to downregulate collagen I expression (Figure 4b). With TGF-β induction, the concentration of collagen I decreased in the culture medium of AACT^KO^ cells and increased in that of AACT^OE^ cells (Figure 4c). Similarly, the concentrations of collagen IV were also detected in the supernatant of HK-2 cells with varied AACT expression. Regardless of TGF-β induction, the concentration of collagen IV decreased in the culture medium of AACT^KO^ cells and increased in that of AACT^OE^ cells (Figure 4e and f). Taken together, AACT promotes the secretion of collagen I and IV during EMT in HK-2 cells. Blocking AACT expression may impede the accumulation of extracellular matrix during renal fibrosis progression.

## 4 Discussion

Untreated LN progresses to end-stage renal disease through progressive fibrosis. The EMT process is widely recognized as a major contributor to myofibroblast accumulation in renal fibrosis, subsequently leading to tubular atrophy [28], [29], [30]. Fortunately, EMT involving tubular epithelial cells is a reversible process. Thus, early identification of targets to combat EMT may provide a promising therapeutic strategy for renal fibrosis. In this study, we reported that AACT was differentially overexpressed in proximal tubule epithelial cells compared with other cell types in kidney across five classes of LN. We observed that the migration ability of these cells was enhanced by AACT overexpression while inhibited when AACT knockout. AACT^OE^ HK-2 cells downregulated the epithelial marker E-cad and upregulated the mesenchymal protein α-SMA. Conversely, MMP2 and MMP9, known to promote cell migration, were upregulation when AACT overexpression. Furthermore, high level of AACT was found to stimulate the secretion of collagen I and IV in HK-2 cells. Taken together, this study identifies AACT as a potential inducer of the EMT process, leading to subsequent fibrosis.

Elevated level of AACT in urine was observed during the transition from acute kidney injury to chronic kidney diseases in animal models and correlated with proliferative LN and other active renal inflammatory diseases in humans [31]. AACT was expressed in the tubular proximal epithelium, as well as in the mesenchyme of nephroblastomas and adult renal cell carcinoma [32]. Likewise, AACT expression was also observed in immunostaining of proximal tubules from control biopsies [33]. Consistently, we found that AACT was predominantly localized in the cytoplasm of tubular epithelial cells of NCs samples. However, there was a marked increase in AACT expression in proximal tubule cells across all LN classes compared with NCs. These findings suggest a potential role for AACT in the inflammatory response associated with kidney damage during the evolution of renal disease from its early- to end-stage.

In addition to AACT, the associations of other cytokines, such as TNF-α, with chronic inflammation or tissue fibrosis have been extensively studied [34], 35], suggesting the complexity of the development of inflammatory diseases such as LN. Although numerous reports have demonstrated the association between AACT and organ fibrosis, the expression pattern of AACT in organ fibrosis remains unclear. The present study illustrated that AACT was differentially expressed in the renal tissues of LN patients, with a notable upregulation in class II of LN and further escalation in class III to V. Therefore, we propose that AACT is not only an early inducer of renal fibrosis but also a biomarker reflecting LN prognosis. In previous studies, the biomarkers for LN disease were systematically investigated, revealing that the urinary AACT level was higher in LN patients with active renal stage compared with other inactive diseases. Additionally, multiple logistic regression analyses identified that AACT was the optimal marker to differentiate active renal from active non-renal disease, suggesting that urinary AACT is a potential biomarker of LN activity [36]. Based on these observations, we hypothesize that the expression of AACT progressively increases in the renal tissues of LN patients with early-stage disease and is subsequently detected in urine as a free form. Consequently, monitoring the aberrant expression of AACT *in situ* within the kidney may provide a more accurate reflection of LN progression than merely detecting urinary AACT.

The relationship between the increasing number of tubular epithelial cells involved in EMT and the decrease in excretory renal function implies that EMT plays a pathogenic role in the advancement of chronic renal diseases. Further evidence supporting this EMT role comes from the observation that reversing EMT leads to improved renal function and decreased mortality rates in a mouse model of crescentic glomerulonephritis [37]. EMT is recognized as an early event in the development of renal fibrosis [38], 39]. The reversal of EMT process holds great significance in the treatment of renal fibrosis. E-cad is a specific intercellular molecule expressed in epithelial cells, and its reduced expression in mesenchymal lineage cells can induce a mesenchymal to epithelial transition [40]. On the contrary, α-SMA is minimally expressed in epithelial cells but abundantly present in mesenchymal cells. Our findings revealed that AACT overexpression downregulated E-cad expression while upregulating α-SMA level in HK-2 cells. In contrast, AACT knockout reduced α-SMA expression compared to wildtype HK-2 cells. Furthermore, AACT upregulated the expression of MMP2 and MMP9, two proteases responsible for cell migration and invasion. Therefore, AACT serves as an upstream regulator in the EMT signaling pathway. Targeting AACT could potentially offer an effective strategy for inhibiting the EMT process during LN development. However, the relationship between AACT expression and organ fibrosis should be further clarified *in vivo*. The detailed signal network of the EMT process regulated by AACT warrants further investigation.

Many studies have established that excessive collagen deposition contributes to EMT and organ fibrosis [41], [42], [43]. Our observations indicate that the levels of collagen I and IV were elevated in the culture medium of AACT^OE^ cells, while they were reduced in the culture medium of AACT^KO^ cells compared with that of AACT^Ctrl^ cells. Hence, the development of AACT-induced renal fibrosis may depend on the following progression: elevated AACT expression determines the levels of factors associated with EMT, enabling epithelial cells to acquire mesenchymal phenotypes. AACT enhances the migration and infiltration abilities of renal epithelial cells via promoting the secretion of MMP2 and MMP9. Ultimately, AACT enhances an excessive deposition of collagen I and IV in renal tissue, thereby accelerating renal fibrosis.

In conclusion, our study provides explicit evidence that AACT is highly expressed in renal tissues from LN patients with classe I to V and preferentially overexpressed in proximal tubules. Significantly, we elucidate the involvement of AACT in the EMT progression of proximal tubule epithelial cells and subsequent renal fibrosis. AACT represents a promising therapeutic target for early-stage renal fibrosis. Due to the complex pathogenesis of renal damage and fibrosis in LN, targeting a single factor alone cannot completely prevent the disease progression. The synergistic effect of AACT with other mediators in inducing renal fibrosis still requires further investigation.

## Supplementary Material

Supplementary Material
