# Supplementary material for: Alpha-1-antichymotrypsin: a potential inducer for epithelial-mesenchymal transition in lupus nephritis
Source: Open Life Sci. 2026 May 20;21(1):20251319. doi: 10.1515/biol-2025-1319 (PMC13211319; doi:10.1515/biol-2025-1319)
Supplement: Supplementary file 1 — Supplementary Material [file j_biol-2025-1319_suppl_001.docx]

**Alpha-1-antichymotrypsin: a potential inducer for** **epithelial-mesenchymal transition in lupus nephritis**

Xiaoyan Huang^2#^, Cuijuan Zhang^3#^, Ming Yang^4^, Xiaoshu Dong^1^, Hui Zhang^5^, Xingjiao Liu^1^, Yi Jiang^1^, Yongfei Wang^7,8^, Yushuang Wei^6^, Bing Yuan^6^, Meiying Wang^1*^

1. Department of Rheumatology and Immunology, Shenzhen Second People’s Hospital, The First Affiliated Hospital of Shenzhen University, Shenzhen, Guangdong Province, 518055, China.
2. Department of Nephrology, Peking University Shenzhen Hospital, Shenzhen, Guangdong Province, 518036, China.
3. Department of Cardiovascular Surgery, First Center of 301 Chinese PLA General Hospital, Beijing, 100853, China.
4. Department of Otolaryngology, Shenzhen People’s Hospital, Shenzhen 518020, China.
5. Department of Laboratory Medicine, Shenzhen Second People’s Hospital, The First Affiliated Hospital of Shenzhen University, Shenzhen, Guangdong Province, 518020, China.
6. Songshan Lake Materials Laboratory, Dongguan, Guangdong Province, 523808, China.
7. School of Medicine and Warshel Institute for Computational Biology, Chinese University of Hong Kong, Shenzhen, Guangdong, China.
8. Department of Paediatrics and Adolescent Medicine, University of Hong Kong, Hong Kong, China.

#These authors contributed equally to this work.

*Corresponding author:

Meiying Wang, M.D, Email: wmy99wmy99@163.com


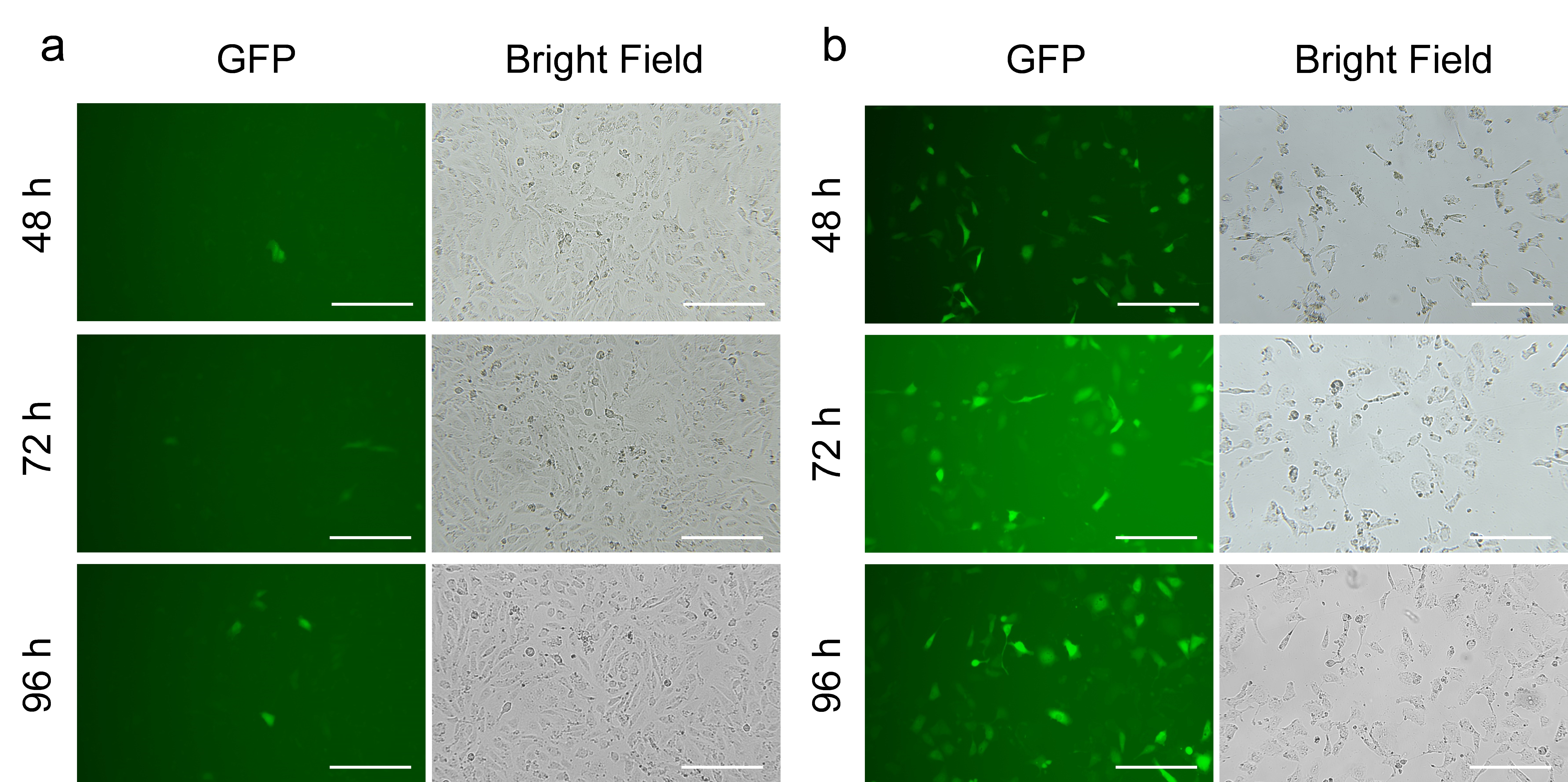


**Supplementary Figure 1. AACT knockout in HK-2 cells.** In vitro transfection results of (a) AACT-Cas9-sgRNA1 and (b) AACT-Cas9-sgRNA2 into HK-2 cells. Scale bar is 50 μm.


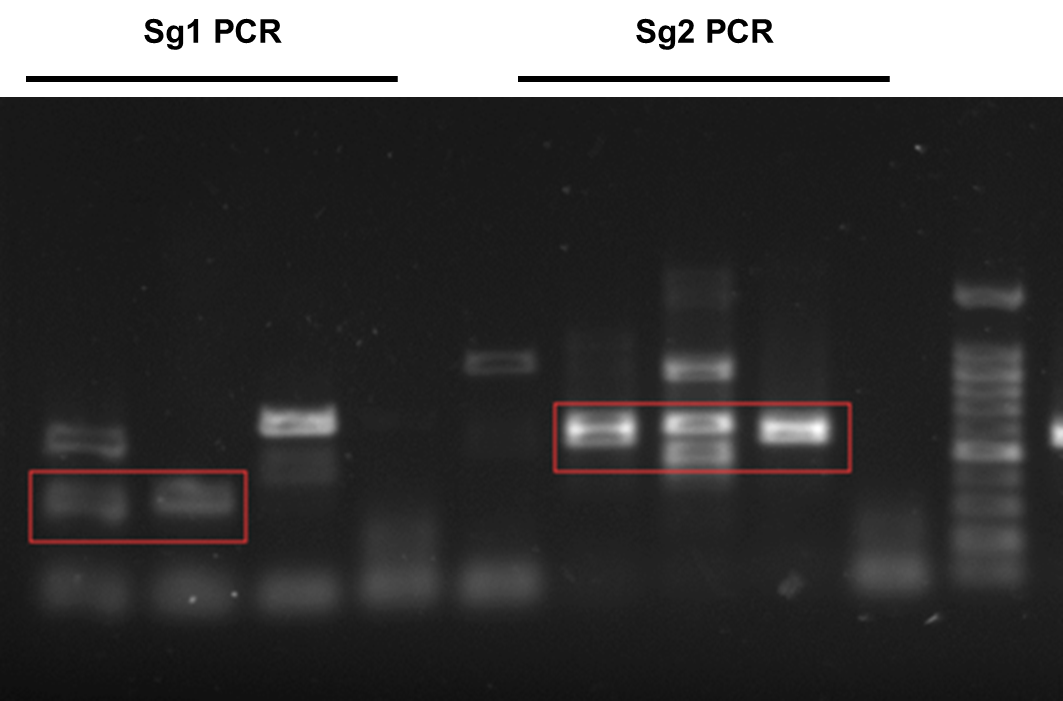


**Supplementary Figure 2. PCR amplified assay showed the cutting efficiency of sgRNA-1 and sgRNA-2.**

**
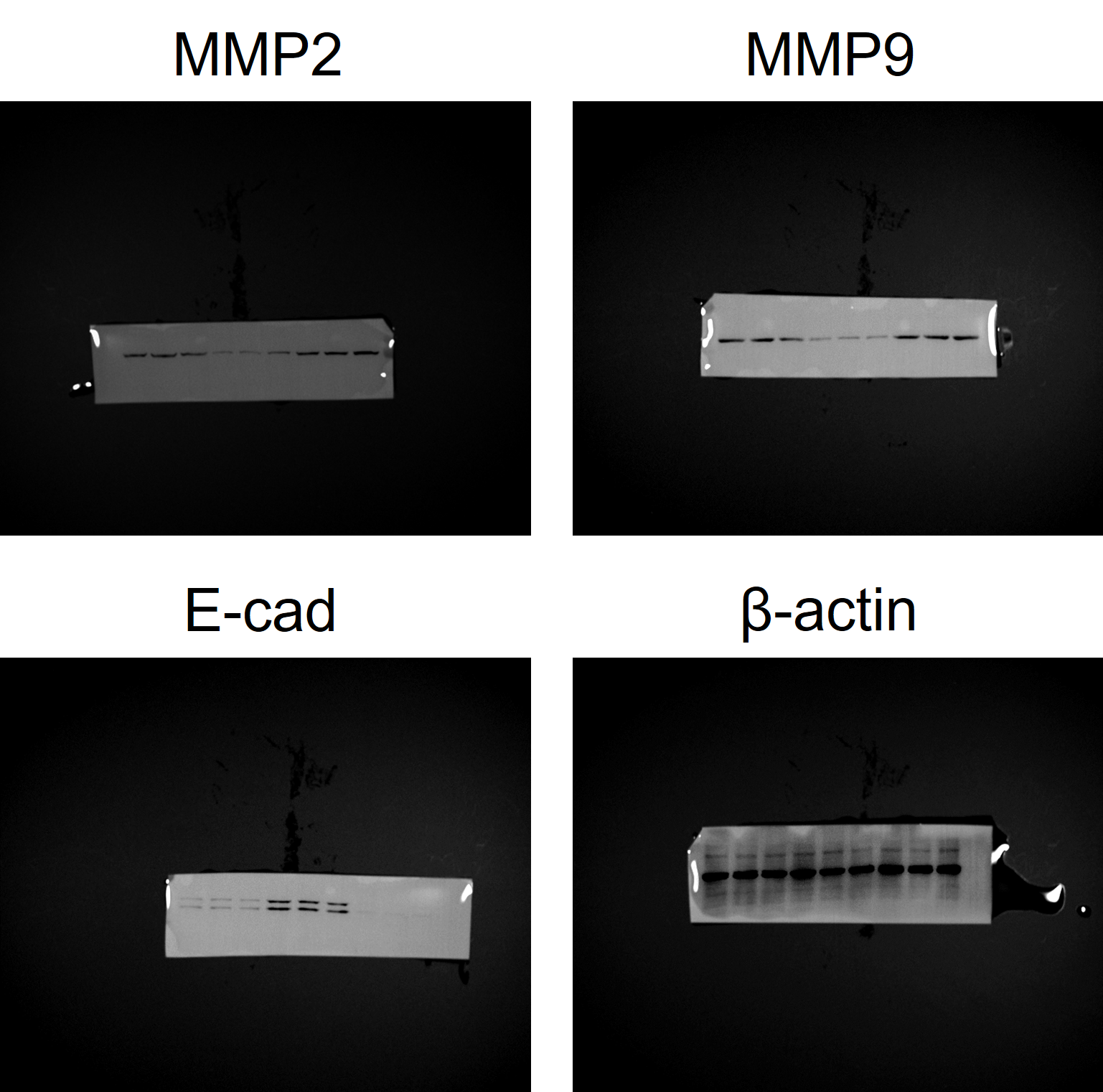
**

**Supplementary Figure 3. Untrimmed images of western blots.**

**Supplementary Table 1. Demographic features and histopathologic findings of LN patients.**

| **Characteristics** | **Patients (n = 42)** | **NCs (n = 6)** |
| --- | --- | --- |
| **Age, year** | | |
| Median (range) | 21 (7-49) | 18 (7-41) |
| ≤18 | 10 (23.8%) | 04 (66.7%) |
| >18 | 32 (76.2%) | 02 (33.3%) |
| **Gender, n (%)** | | |
| Females | 34 (81.0%) | 03 (50.0%) |
| Males | 08 (19.0%) | 03 (50.0%) |
| **ISN/RPS lupus class, n (%)** | | |
| I | 06 (14.3%) | No |
| II | 06 (14.3%) | No |
| III | 06 (14.3%) | No |
| IV | 06 (14.3%) | No |
| V | 06 (14.3%) | No |
| III+V | 06 (14.3%) | No |
| IV+V | 06 (14.3%) | No |
| **Light microscopy, n (%)** | | |
| Cellular/fibrocellular crescent | 11 (26.2%) | No |
| Fibrous crescent | 07 (16.7%) | No |
| Increased mesangial matrix | 31 (73.8%) | No |
| Mesangial hypercellularity | 40 (95.2%) | No |
| Increased matrix and Mesangial hypercellularity | 25 (59.5%) | No |
| Endocapillary hypercellularity | 21 (50.0%) | No |
| **Glomerulosclerosis, n (%)** | | |
| Absent | 14 (33.3%) | No |
| 1-9% | 12 (28.6%) | No |
| 10-25% | 11 (26.2%) | No |
| >25% | 11 (26.2%) | No |
| Activity score ≥8 (score is out of 24) | 07 (16.7%) | No |
| Chronicity scores ≥6 (score is out of 12) | 09 (21.4%) | No |
| **Electron Microscopy, n (%)** | | |
| Mesangial deposits | 39 (92.9%) | No |
| Subendothelial deposits | 34 (81.0%) | No |
| Subepithelial deposits | 31 (73.8%) | No |
| Intra-membranous deposits | 10 (23.8%) | No |
| **Foot process effacement** | | |
| Mild | 12 (28.6%) | No |
| Moderate-severe | 27 (64.3%) | No |
| Tubuloreticular inclusions (TRI) | 26 (61.9%) | No |
| Thrombotic microangiopathy (TMA) | 02 (4.76%) | No |
